# High adherence to national IPC guidelines as key to sustainable VRE control in Swiss hospitals: a cross-sectional survey

**DOI:** 10.1186/s13756-022-01051-9

**Published:** 2022-01-28

**Authors:** Danielle Vuichard-Gysin, Rami Sommerstein, Andreas Kronenberg, Niccolò Buetti, Marcus Eder, Vanja Piezzi, Céline Gardiol, Matthias Schlegel, Stephan Harbarth, Andreas Widmer, for Swissnoso

**Affiliations:** 1 Swiss Center for Infection Prevention, Swissnoso, Bern, Switzerland; 2 Infectious Diseases and Hospital Epidemiology, Thurgau Hospital Group, Muensterlingen and Frauenfeld, Switzerland; 3 Department of Infectious Diseases, Bern University Hospital, University of Bern, Bern, Switzerland; 4 Department of Health Sciences and Medicine, University of Lucerne, Lucerne, Switzerland; 5 Institute for Infectious Diseases, University of Bern, Bern, Switzerland; 6 Infection Control Program and WHO Collaborating Centre on Patient Safety, University of Geneva Hospitals and Faculty of Medicine, Geneva, Switzerland; 7 Federal Office of Public Health, Bern, Switzerland; 8 Division of Infectious Diseases and Hospital Epidemiology, Cantonal Hospital St. Gallen, St. Gallen, Switzerland; 9 Division of Infectious Diseases and Hospital Epidemiology, University of Basel Hospitals and Clinics, Basel, Switzerland

**Keywords:** Vancomycin resistant *Enterococcus faecium*, Surveillance, Infection prevention and control, Admission screening, Contact precautions, Outbreak, Acute care

## Abstract

**Background:**

Vancomycin resistant enterococci (VRE) are on the rise in many European hospitals. In 2018, Switzerland experienced its largest nosocomial VRE outbreak. The national center for infection prevention (Swissnoso) elaborated recommendations for controlling this outbreak and published guidelines to prevent epidemic and endemic VRE spread. The primary goal of this study was to evaluate adherence to this new guideline and its potential impact on the VRE epidemiology in Swiss acute care hospitals.

**Methods:**

In March 2020, Swissnoso distributed a survey among all Swiss acute care hospitals. The level of adherence as well as changes of infection prevention and control (IPC) strategies in the years 2018 and 2019 after publication of the national guidelines were asked along with an inventory on VRE surveillance and outbreaks.

**Results:**

Data of 97/146 (66%) participants were available, representing 81.6% of all acute care beds operated in Switzerland in 2019. The vast majority—72/81 (88%) responding hospitals—have entirely or largely adopted our new national guideline. 38/51 (74.5%) hospitals which experienced VRE cases were significantly more likely to have changed their IPC strategies than those 19/38 (50%) hospitals without VRE cases *p* = 0.017). The new IPC guidelines included (1) introduction of targeted admission screening in 89.5%, (2) screening of close contacts of VRE cases in 56%, and (3) contact precaution for suspected VRE cases 58% of these hospitals. 52 (54%) hospitals reported 569 new VRE cases in 2018 including 14 bacteremia, and 472 new cases in 2019 with 10 bacteremia. The ten largest outbreaks encountered between 2018 and 2019 included 671 VRE cases, of which most (93.4%) consisted of colonization events, 29 (4.3%) infections and 15 (2.2%) bacteremia.

**Conclusion:**

Wide adoption of this VRE control guideline seemed to have a positive effect on VRE containment in Swiss acute care hospitals over two years, even if its long-term impact on the VRE epidemiology remains to be evaluated. Broad dissemination and strict implementation of a uniform national guideline may therefore serve as model for other countries to fight VRE epidemics on a national level.

**Supplementary Information:**

The online version contains supplementary material available at 10.1186/s13756-022-01051-9.

## Introduction

Vancomycin resistant enterococci (VRE) are increasing in many European countries. In their last annual epidemiological report, the European Center for Disease Control and Prevention has issued a statement of concern [1]. In 2018, the Federal Office of Public Health (FOPH) commissioned Swissnoso, the Swiss Center for Infection Prevention, to investigate a large country-wide nosocomial outbreak of VRE affecting several hospitals with spill-over into many other cantons [2]. This investigation revealed gaps in national surveillance and communication between hospitals and public health authorities. This prompted the Swiss Federal office of public health to making reporting of VRE clusters mandatory [3, 4].

There is no evidence from any European country, that current strains of VRE and clonal complexes that are endemic in healthcare settings, have started to circulate in the community [5]. This contrasts with ESBL-producing *E. coli*, which are introduced into healthcare settings through widespread transmission in the community [5]. Lack of adherence to infection control measures, including extensive environmental contamination and high antibiotic pressure, seem to be major drivers of in-hospital VRE acquisition and transmission [6, 7]. VRE transmission mainly results in colonization of the patient, but rarely causes invasive infections such as bacteremia [8, 9]. Therefore, transmission in the absence of systematic screening policies will remain undetected allowing VRE to spread within and across healthcare facilities [10–12]. That strict adherence to contact precautions (CP) can prevent local transmission of multidrug-resistant organisms, including VRE, was recently demonstrated by a local Swiss healthcare institution [13]. However, compared to other European countries such as France [14], Germany [15], or the Netherlands [16], Switzerland had no uniform national guidelines for prevention and control of VRE transmission in hospitals. Therefore, the national center for infection prevention (Swissnoso) issued new guidelines in September 2018, and updated them in December 2019 [17, 18], based on scientific evidence and existing international and national publications [19, 20]. The goal was not only to eliminate the clusters responsible of the outbreak, but to largely control VRE at the national level. In addition to the mandatory reporting, Swissnoso made use of the systematic surveillance implemented by the national center of antibiotic resistance (ANRESIS) and started publishing quarterly data on new VRE cases reported to this platform (www.anresis.ch) [21].

The main objective of this study was to evaluate the adherence with the new national VRE control guideline and its potential impact on the VRE epidemiology in Swiss acute care hospitals. To improve our national VRE surveillance strategy in the future, our secondary goal was to externally validate the VRE data collected by ANRESIS.

## Methods

In March 2020, a 34-item questionnaire was sent to 146 infection control professionals at 204 acute-care institutions providing inpatient care. Long-term care facilities, nursing homes, and psychiatric institutions were excluded. Non-responding institutions were reminded three times by e-mail.

A questionnaire from a survey conducted in 2018 was updated to meet the current needs [12]. The questionnaire was pre-tested by several infection control nurses and physicians for comprehensibility and time needed. The survey was translated in the three official languages and eventually shared through the online platform SurveyMonkey®. Participants were asked to provide answers for their institutions as well as for others they provided IPC services. If respondents indicated that they were answering for more than one center, they were required to indicate whether those answers were the same or different with respect to the IPC measures. If they were different, we asked them to complete the survey separately for each center.

We collected information on local VRE control strategies including self-reported level of adoption of the guidelines, recent changes in IPC measures, presence of local standards for VRE screening and preventive CP, and whether there were any barriers to implement specific recommendations. In addition, we asked hospitals to provide their total number of new VRE cases detected by year and the proportion of VRE positive blood cultures detected. VRE was defined as *Enterococcus faecium* with phenotypical amoxicillin- and vancomycin-resistance on routine susceptibility testing. VRE cases were considered “new” if the first detection occurred at the corresponding institution. For hospitals reporting outbreaks, we requested a description of the largest outbreak including detailed information such as the number of isolates detected overall and in blood cultures, resistance phenotypes, and the mode of detection (culture and/or PCR). In addition, we asked them to provide a rough estimate of the clonal relatedness of isolates, e.g., whether < 50%, 50–75% or > 75% were considered clonally related. An outbreak was defined as a situation with an unexpected accumulation of ≥ 3 cases with a positive laboratory test result for VRE from either a clinical sample or screening specimen and with an epidemiological link (temporal, local). Molecular genetic detection was not necessarily required to meet the definition. Survey results were analyzed respondent-based. The survey allowed for multiple attempts to enter data by the same hospital. In case of conflicting answers between different versions, the latest data entry was considered. In addition, the first author contacted the participants by e-mail and asked to verify their entries.

For the preparation of this manuscript, the authors followed the revised standards for quality improvement reporting excellence (SQUIRE 2.0) [22].

### Statistical analyses

Data were exported from the online platform to an Excel® spread sheet, checked for accuracy, cleaned, and imported for descriptive analyzes into SPSS® [25]. Categorical data were compared by Chi-square test. Results were either stratified by hospital size or by cantons, as deemed suitable.

### Validation of the ANRESIS database

The Swiss Centre for Antibiotic Resistance (ANRESIS) is a national surveillance system. Participating clinical microbiology laboratories report their antimicrobial susceptibility test results anonymously. The laboratories are well distributed across the different territories in Switzerland and represent isolates from tertiary-care hospitals, as well as cantonal and private laboratories. All antimicrobial resistance data are derived from routinely performed analyses and include isolates from sterile as well as non-sterile sites. Resistance reports are publicly available with data aggregated by predefined regions [21].

One of the co-authors (AK) performed an extraction of all VRE cases reported between January 1st, 2018, and December 31st, 2019, and classified them by canton. The surveillance period covered 81% and 89% of annual patient-days (PDs) for 2018 and 2019, respectively. Using the annual VRE numbers from the survey and the ANRESIS database for 2018 and 2019, which were both aggregated at the cantonal level, we created Bland–Altman diagrams for each, bacteremia and non-bacteremia, separately [23] and estimated the correlation coefficient. We defined a priori that agreement between the two datasets was high, if 90% or more of the data points (indicating the difference between the numbers reported in the survey and those retrieved from ANRESIS) clustered around the mean of the differences within two standard deviations of the mean. Agreement was defined as moderate, if at least 75% (but < 90%) of the data points lie within the 95% limits of agreement. Each data point represents the data of a canton.

In addition, we evaluated agreement between the two surveillance tools by visual inspection of the bar charts showing the absolute numbers of new VRE cases by years and cantons separately for blood cultures (bacteremia) and other clinical samples (non-bacteremia) according to Smith et al*.* [24].

## Results

### Characteristics of participating hospitals

**Fig. 1:**
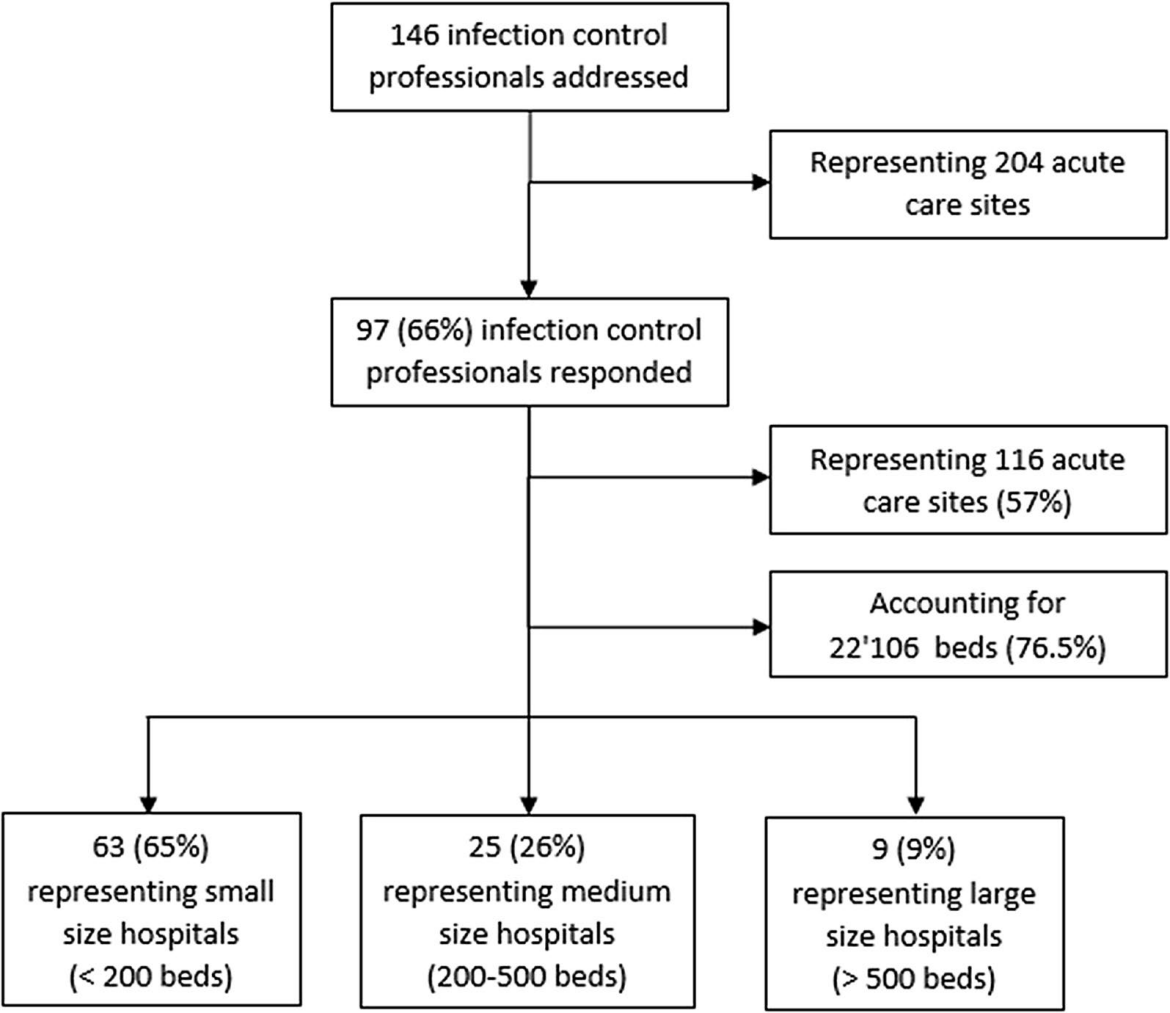
Flow chart of survey respondents and representing institutions

We addressed a total of 146 infection control professionals or hospital epidemiologists who are responsible for 204 acute care sites. Of these, 97 (66%) participated in the survey, 81 representing a single institution and 16 being responsible for several sites, translating into a total of 116 acute care sites or institutions across 24 cantons. The participants indicated to serve a total of 22,106 beds, which is 81.6% of all acute care beds operated in Switzerland in 2019 according to the Swiss Federal Office of Public Health [25]. The participants represented 63 (65%) small (< 200 beds), 25 (26%) medium (200–500 beds) and 9 (9%) large (> 500 beds) hospitals (Fig. 1).

### Local VRE control strategies

**Fig. 2:**
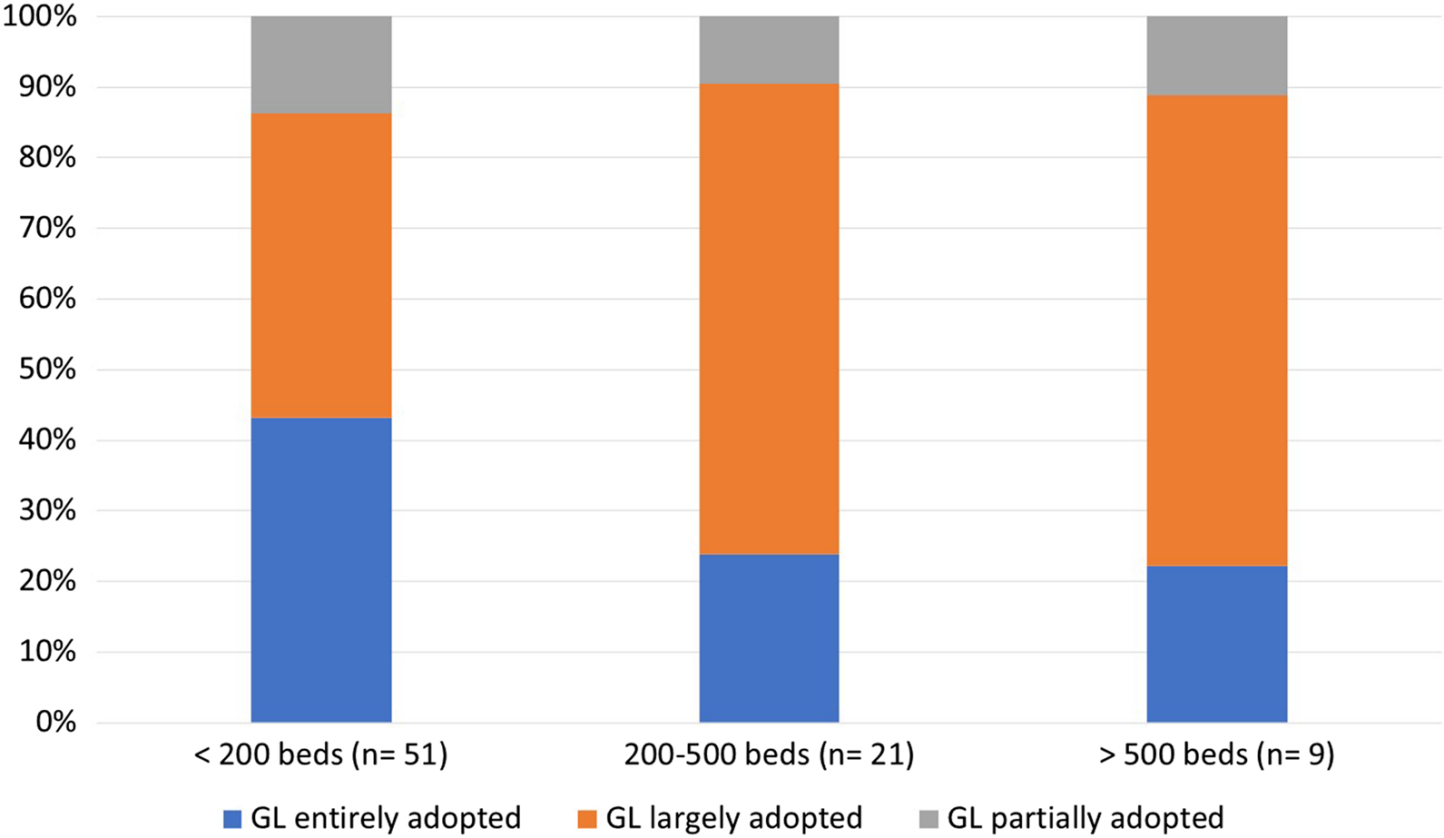
Self-reported compliance with Swissnoso guideline (GL)—according to hospital size

**Fig. 3:**
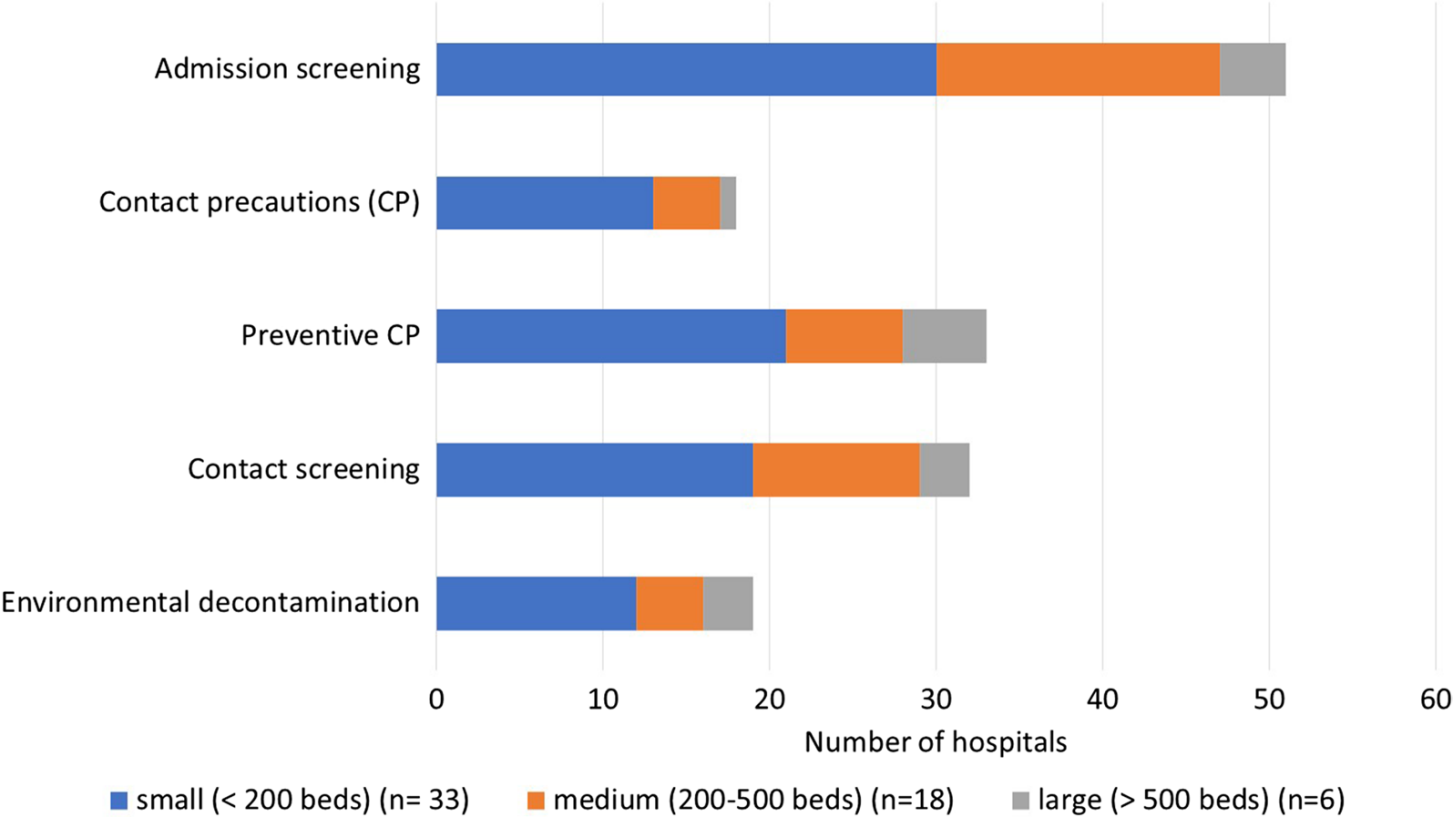
Reported infection prevention and control measures introduced since beginning of 2018, stratified by hospital size

Overall, 88% (72/81) of the responding IPC professionals stated that they had fully or largely adopted the Swissnoso guideline in their institutions. Stratified by hospital size, these were 86% (44/51), 90% (19/21), and 89% (8/9) of all responding small, middle and large hospitals, respectively (Fig. 2). Of 90 respondents, 57 (63%) indicated an intensification of their infection prevention and control (IPC) strategies since beginning of 2018. Among the different hospital sizes, the proportion of medium-sized hospitals that enhanced their VRE control measures was highest at 78.3%, compared with 57.9% (small hospitals) and 66.7% (large hospitals). However, this difference was not statistically significant (Table 1). In contrast, there was a significant association between VRE epidemiology and intensification of IPC management: Of 51 hospitals which noted VRE cases, 38 (74.5%) enhanced their VRE control measures within the last two years compared to 19 of 38 hospitals (50.0%) with no VRE cases (*p* = 0.017) (Table 1). Changes in IPC strategies included introduction of admission screening (51/57, 89.5%), contact precaution of confirmed VRE cases (18/57, 31.6%), preventive CP for suspected VRE cases (33/57, 58%), screening of close contacts of VRE cases (32/57, 56.1%), and intensification of environmental decontamination (19/57, 33.3%). Among the different hospital sizes, the proportion of hospitals that introduced admission screening was especially high in small- and middle-sized hospitals (90.9% and 94.4%, respectively), while introduction of CP for confirmed VRE cases was a novum for 39.4% of small hospitals and introduction of preventive CP for VRE suspects was new for 83.3% of large hospitals (Fig. 3). As far as the knowledge of the guideline was concerned, 77 (88.5%) were aware of the 2019 update and 7 (8%) were aware of at least the original Swissnoso recommendation issued in 2018. A total of 87 IPC professionals responded to this question.

**Table 1:** Enhancement of VRE infection control measures since 2018 according to hospital size and experience with VRE cases

	VRE control measures intensified	*p* value
Small hospitals (< 200 beds) (n = 57)	33 (57.9%)	0.225
Medium hospitals (200–500 beds) (n = 23)	18 (78.3%)	
Large hospitals (> 500 beds) (n = 9)	6 (66.7%)	
Has never had any VRE cases (n = 38)	19 (50.0%)	**0.017**
Had already VRE cases (n = 51)	38 (74.5%)	

A* p*-value of < 0.05 was considered statistically significant

The inventory on measures for VRE prevention and control already in place in hospitals according to the Swissnoso recommendations revealed a high compliance with the recommended measures across all hospital sizes. With respect to specific recommendations for targeted admission screening, most small, medium, and large hospitals indicated to screen all transfers from hospitals in other cantons with an ongoing VRE epidemic as well as direct transfers from hospitals abroad. The proportion of hospitals with a recommendation to screen patients with a previous hospital stay abroad or for transfers from high-risk wards such as haemato-oncology was lower, whereas the proportion of hospitals with a written standard for screening patients with a history of VRE carriage markedly varied according to hospital size with the smallest proportion among medium-sized hospitals (Additional file 1: Fig. S1a). The survey further revealed that > 90% of hospitals had a written standard for preventive CP. The weights put on specific recommendations, however, varied in a similar way as for admission screening. While most hospitals indicated to have recommendations for preventive CP for VRE contacts, transfers from other cantons with a VRE epidemic and for direct transfers from abroad, the proportion of respondents was lower regarding recommending preventive CP for patients with a previous stay abroad, transfer from another ICU with unknown epidemiologic situation or a high-risk ward such as haemato-oncology (Additional file 1: Fig. S1b). Out of 85 respondents, 13 (15.3%) reported difficulties in implementing all recommendations. Nine reservations were specified and related to implementation of CP for VRE cases (2/84, 2.1%), adoption of preventive CP for VRE contacts (3/84, 3.1%) and admission screening (4/85, 4.1%).

### VRE epidemiology

**Fig. 4:**
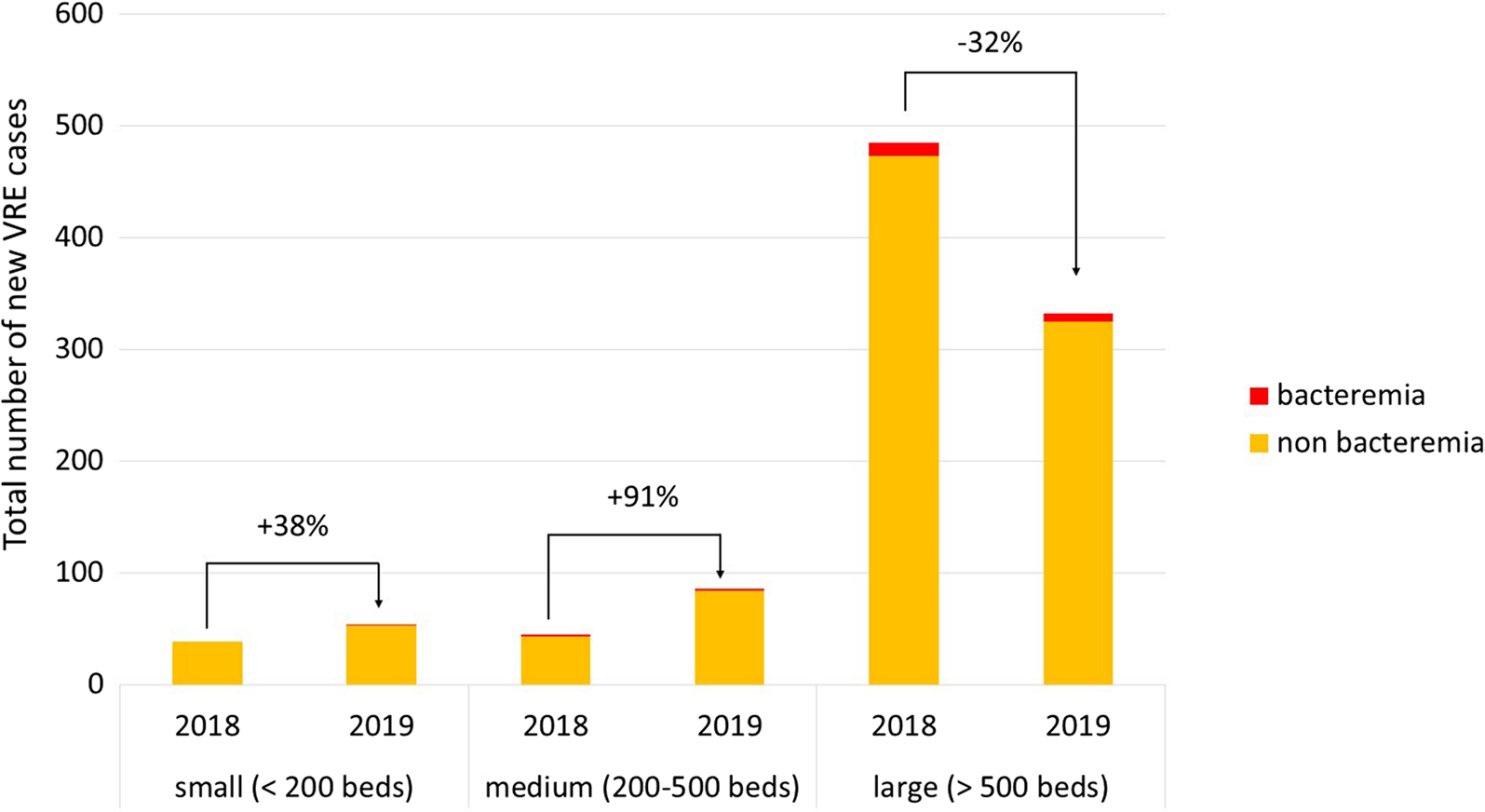
Total number of new VRE cases detected per year (2018–2019) according to size of hospitals

Between January 1, 2018, and December 31, 2019, 52 (54%) participants reported a total of 1041 new VRE cases, 569 new VRE cases in 2018 and 472 new cases in 2019. The total number of new VRE cases reported per year differed by hospital size, with small and medium-sized hospitals reporting increases of 15 and 41 cases (plus 38%-points and plus 91%-points, respectively) in 2019, whereas the number of new VRE cases detected in large hospitals decreased substantially by 153 cases (minus 32-% points) in 2019 compared with 2018 (Fig. 4). The numbers reported by hospitals also varied considerably between the federal cantons (Additional file 1: Fig. S2). Bern was the canton most affected by the large VRE outbreak as has been previously described [2]. From 2018 to 2019, there was an overall increase of VRE detection by means of admission screening with a strong proportional increase from 32 to 89% among small hospitals (Additional file 1: Fig. S3). The Proportions of bacteremia remained low in both years with 14 (2.5%) in 2018 and 10 (2.1%) in 2019.

### VRE outbreaks

A total of 14 facilities reported at least one outbreak (3 of each small and medium-sized hospitals and 8 large hospitals), 8 in 2018 and 6 in 2019, resulting in a total of 16 outbreaks over the two years. Of all outbreaks reported, the ten largest outbreaks were described in more detail and characterized by a median duration of 14 weeks (interquartile range, IQR 6.5–37.0); three took place each on intensive care units, on surgical wards, and internal medicine wards, two occurred on neonatology, one on geriatrics, and one outbreak affected several wards not otherwise specified. They comprised a total of 671 laboratory confirmed VRE cases (64.5% of all new VRE cases reported). The vast majority of cases—namely 627 (93%)—represented colonizations, while only 29 (4.3%) and 15 (2.2%) cases were affected by infection and bacteremia, respectively. Most VRE isolates were Teicoplanin susceptible and/or harbored the *vanB* gene (n = 588, 86.6%). Further details are listed in Table 2. In 7 of 10 outbreaks at least 50% of all detected isolates were genotyped, and more than 75% of isolates were considered clonal in 6 of 10 outbreaks. 75 of 82 respondents (91%) confirmed their knowledge about the recently introduced mandatory reporting of VRE outbreaks issued by the Federal Office of Public Health.

**Table 2:** Hospitals describing their largest outbreak

	Total	2018	2019
*Total number of outbreaks*	10	5	5
Median duration of outbreak in weeks (IQR)	14 (6.5–37.0)	8 (4–58)	20 (12–43)
*Involved wards*			
ICU, n (%)	3 (20.0)	1 (13.0)	2 (29.0)
Surgical ward, n (%)	3 (20.0	2 (25.0)	1 (14.0)
Hemato-oncology, n (%)	2 (13.3)	1 (13.0)	1 (14.0)
Internal medicine, n (%)	3 (20.0)	2 (25.0)	1 (14.0)
Neonatology, n (%)	2 (13.3)	2 (25.0)	0
Geriatric ward, n (%)	1 (6.7)	0	1 (14.0)
Several wards (not specified), n (%)	1 (6.7)	0	1 (14.0)
Involved VRE cases, median (IQR)	11 (7–28)	11 (6–274)	12 (7–38)
Total VRE cases	671	570 (85.0)	101 (15.0)
VRE bacteremias, n (%)	15 (2.2)	10 (2.0)	5 (5.0)
VRE infection, n (%)	29 (4.3)	15 (3.0)	14 (14.0)
VRE detected by screening, n (%)	627 (93.4)	545 (96.0)	82 (81.0)
Teicoplanin-resistant VRE isolates, n (%)	91 (13.4)	71 (12.0)	20 (19.0)
Teicoplanin-susceptible VRE isolates, n (%)	588 (86.6)	502 (88.0)	86 (81.0)

*Annotation*

If several specific wards were affected in the same outbreak, each ward counted separately

### Validation of the ANRESIS database

**Fig. 5:**
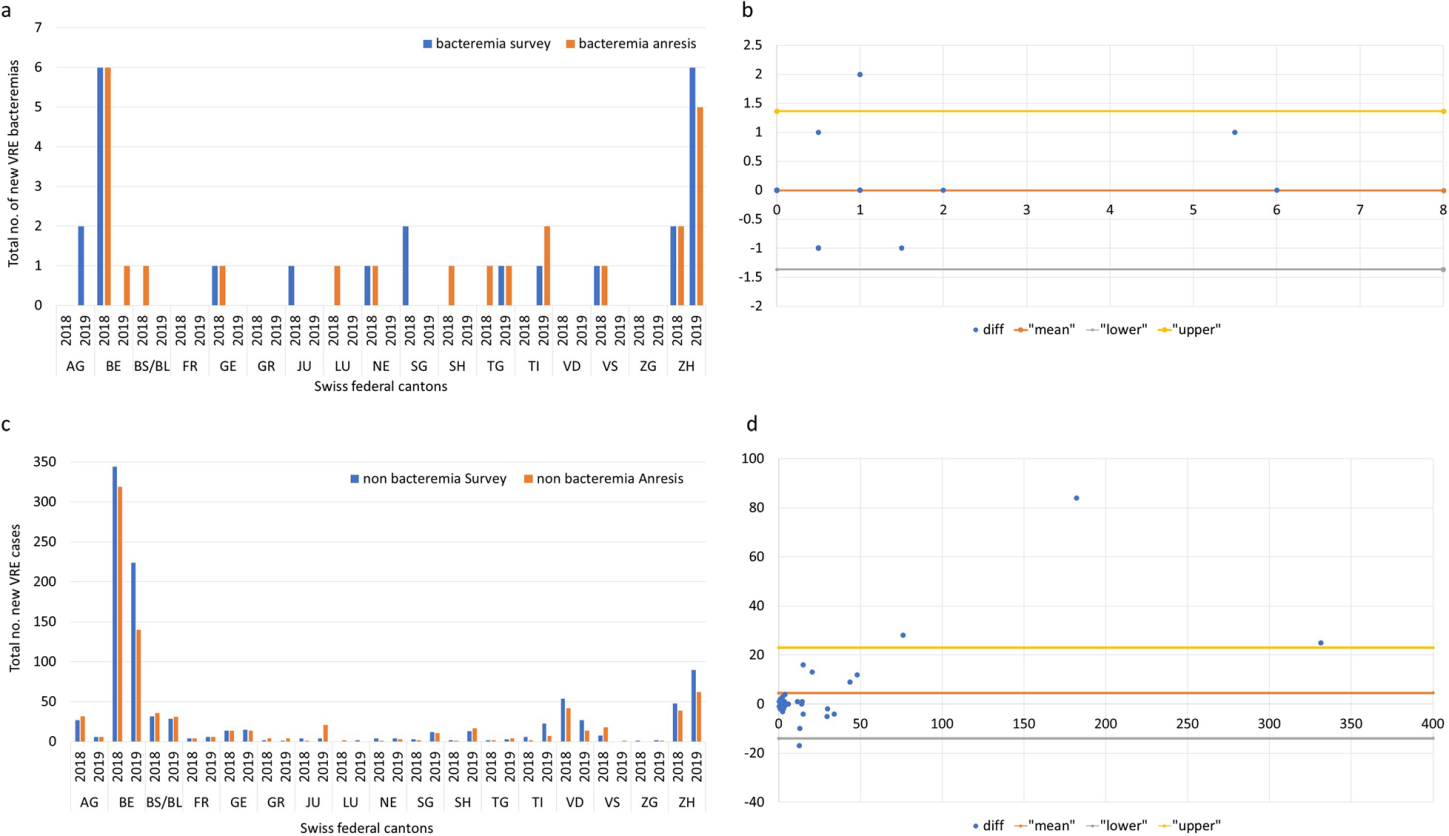
Validation of the ANRESIS surveillance for invasive and non-invasive VRE isolates. **a** Total number of new VRE bacteremia cases reported by hospitals and recorded by ANRESIS per canton and year (2018–2019). The blue bars correspond to the numbers reported in the national survey, the orange bars correspond to the numbers collected by ANRESIS. Only cantons with at least one VRE case have been considered. **b** Bland–Altman Plots for the level of agreement between the two surveillances in reporting cases with VRE bacteremia. “diff” indicates the absolute difference in reported cases between the two systems, “lower” indicates lower limit of agreement, “upper” indicates upper limit of agreement, while “mean” indicates the mean of differences, also called bias. **c** Total number of new non-bacteremia VRE cases reported by hospitals and recorded by ANRESIS per canton and year (2018–2019). The blue bars correspond to the numbers reported in the national survey, the orange bars correspond to the numbers collected by ANRESIS. Only cantons with at least one VRE case have been considered. **d** Bland–Altman Plots for the level of agreement between the two surveillances in reporting non-bacteremia VRE cases. “diff” indicates the absolute difference in reported cases between the two systems, “lower” indicates lower limit of agreement, “upper” indicates upper limit of agreement, while “mean” indicates the mean of differences, also called bias

Visual inspection of the bar charts showing the absolute numbers of new VRE cases by year and canton for bacteremia (Fig. 5a) and non-bacteremia (Fig. 5c) showed good concordance between the two reporting systems (survey and ANRESIS). Comparison of the number of new VRE isolates as reported by survey participants and recorded in the ANRESIS database stratified by canton showed a strong correlation for bacteremia (correlation coefficient of 0.88) and non-bacteremia (correlation coefficient 0.98) isolates. Agreement between the two reporting systems was high with 32/34 (94%) measured differences in bacteremia and 33/34 (97%) measured differences in non-bacteremia lying between the 95% limits of agreement, which is above the pre-defined value of 90% (Fig. 5b, d). The outlier in Fig. 5d with a difference of 84 belongs to the canton Bern that was most affected by the largest outbreak Switzerland has ever experienced.

## Discussion

This survey reflects the first comprehensive inventory on the strategies to prevent and control the spread of VRE within and across hospitals in Switzerland and an update of the current VRE epidemiology including the detailed description of the ten largest outbreaks. To our surprise, 88% (72/81) of the responding IPC professionals indicated to have fully or largely adopted the Swissnoso guideline in their institutions. There was no significant difference between the size of hospitals regarding guideline adoption. However, we observed that hospitals who had already encountered VRE cases were significantly more likely to have recently enhanced their VRE control strategy. Our results show that a high proportion of Swiss acute care hospitals have stringent measures in place to control the spread of VRE, thus exhibiting a high level of compliance with the national recommendations published by Swissnoso. Furthermore, we did not encounter significant barriers to the implementation of these guidelines. A national survey across German hospitals revealed that most of them had guidelines in place to prevent transmission of and/or infections with methicillin-resistant *Staphylococcus aureus* (MRSA) (99%), multidrug-resistant Gram-negative pathogens (96%) and *Clostridioides difficile* (96%) [26]. Results on adherence to recommendations for prevention and control of VRE transmission were not reported. Except for specific outbreak containment measures that have been previously addressed by several co-authors of this manuscript [12], we did not identify any other publications assessing adherence to VRE control guidelines at national level, at least not in European countries. This work could therefore serve as a good example of a rational approach to assessing IPC compliance beyond Switzerland, applied also to other multidrug-resistant organisms.

We have previously suggested that varying implementation of measures to control the spread of multi-drug resistant organisms in hospitals and different applications of the concept of CP [27] are potential reasons why evidence from studies on the effectiveness of these measures are largely lacking [28]. Compared to countries that resigned from strict VRE containment a while ago [29], our data suggest, that most cantons in Switzerland are successful in controlling local transmission and stopping the inter-cantonal spread of VRE between hospitals due to measures such as admission screening and preventive CP.

Compared to previously published data from Switzerland [12], the number of new VRE cases detected per year, however, has more than doubled. In addition to expanded admission screening in small and medium hospitals, the main reason for this sharp increase in newly diagnosed VRE cases was the large-scale outbreak in the Canton of Bern and surrounding regions, which continued into 2019 and spread to the north-east of Switzerland. Despite this increase in VRE cases, the proportion of invasive isolates remains at a low level. This is in contrast with surveillance data from neighboring countries [30], which show a substantial increase in VRE bloodstream infections, causing a completely different VRE burden. This lower rate of bacteremia could be an indication that our strict measures have an overall containment effect and that the dissemination of VRE in Swiss hospitals has not yet reached an endemic level where intensive control strategies become ineffective. After all, we have seen a sharp decline in VRE cases in large hospitals which were responsible for the main VRE burden.

Previous work has indicated that certain clones of *E. faecium* have a particular propensity for causing nosocomial outbreaks [31, 32]. Thus, even if our results indicate that ANRESIS is a valuable passive surveillance tool, systematic collection of sequencing data may be the crucial next step for the national prospective VRE surveillance in Switzerland and may further contribute to the knowledge about the effectiveness of specific interventions.

Multi-locus sequence typing, pulsed-field gel electrophoresis, and amplified fragment length polymorphism have been proven to be valuable and cheap methods to establish clonality between isolates in different outbreak investigations [33]. However, for bacteria with a more complex genetical diversity such as VRE, whole genome sequencing (WGS) seems to be superior to the conventional typing methods due to its higher discriminative power [34–36]. WGS may also enhance understanding of intra- and inter-regional spread provided that data are collected at the national level which is the case for example in Denmark [37].

One of the strengths of our work is the comprehensiveness of the survey addressed to a broad IPC community. The results of which enabled to accurately describe the specific IPC measures established in Swiss acute hospitals and the current VRE epidemiology.

Limitations include the moderate response rate, which was potentially related to the ongoing COVID-19 pandemic with a shift of resources toward outbreak management. In addition, the granularity of reporting VRE cases by canton can be criticized, since canton-level data may be difficult to be interpreted due to heterogeneity in terms of number and size of hospitals, case mix and total population. Nevertheless, in a federal state with competent public health authorities in each canton, these figures can help to better understand the local epidemiology and guide appropriate IPC measures. Finally, the period of two years for the comparison of observations is too short to adequately validate the ANRESIS VRE reporting and determine whether the bias and agreement observed are consistent.

## Conclusion

The VRE landscape in Swiss acute hospitals has changed significantly in recent years, with an upward trend similar to many other countries in Europe. Nevertheless, the high adherence to the national VRE guidelines gives hope that this negative trend can be halted for the time being. ANRESIS has proven to be a valuable monitoring tool to inform hospitals about the epidemiological situation in other cantons and may therefore serve as additional help to guide local IPC measures.

## Supplementary Information

**Additional file 1.** This file contains the supplementary figures S1a. and b., S2 and S3.

## Data Availability

The datasets generated and/or analyzed during the current study are available from the corresponding author upon reasonable request.
