# Supplementary material for: High adherence to national IPC guidelines as key to sustainable VRE control in Swiss hospitals: a cross-sectional survey
Source: Antimicrob Resist Infect Control. 2022 Jan 28;11:19. doi: 10.1186/s13756-022-01051-9 (PMC8795934; doi:10.1186/s13756-022-01051-9)
Supplement: Supplementary file 1 — Additional file 1. This file contains the supplementary figures S1a. and b., S2 and S3. [file 13756_2022_1051_MOESM1_ESM.docx]

High adherence to national IPC guidelines as key to sustainable VRE control in Swiss hospitals: a cross-sectional survey.

Danielle Vuichard-Gysin^1,2^, Rami Sommerstein^1,3,4^, Andreas Kronenberg^5^, Niccolò Buetti^6^, Marcus Eder^1^, Vanja Piezzi^3^, Céline Gardiol^7^, Matthias Schlegel^8^, Stephan Harbarth^6^*, Andreas Widmer^9^* for Swissnoso

**Supplementary figures:**

**Figures S1a and b. Measures already in place to prevent VRE transmission in hospitals.**

**S1a. Proportion of hospitals with a written standard for admission screening including the following items as stated in the Swissnoso recommendations. Answers are stratified according to hospital size (n = 83 respondents).**

Annotation: prev = previous

**S1b. Proportion of hospitals with a written standard for preventive contact precautions including the items as stated in the Swissnoso recommendations. Answers are stratified according to hospital size (n= 77 respondents).**

Annotation: hem-onc= hemato-oncology; ICU= intensive care unit; prev = previous

**Figure S2. Total number of new VRE cases reported by hospitals per canton and year (2018-2019)**

**Figure S3. Proportion of VRE detection by means of admission screening.**
